# Weathering Tests on Raw and Consolidated Vicenza Stone

**DOI:** 10.3390/ma17143541

**Published:** 2024-07-17

**Authors:** Ilaria Capasso, Abner Colella, Fabio Iucolano

**Affiliations:** 1 Department of Engineering and Geology, University of Chieti-Pescara “G. d’Annunzio”, Viale Pindaro 42, 65122 Pescara, Italy; 2 DiSTAR, Department of Earth Sciences, Environment and Resource, University of Naples Federico II, Via Vicinale Cupa Cintia 21, 80126 Naples, Italy; abner.colella@unina.it; 3 ACLabs—Applied Chemistry Labs, Department of Chemical, Materials and Industrial Production Engineering, University of Naples Federico II, P.le Tecchio 80, 80125 Naples, Italy; fabio.iucolano@unina.it

**Keywords:** Vicenza stone durability, inorganic consolidants, salt crystallization, acid rain simulation, accelerated weathering

## Abstract

The preservation of cultural heritage, particularly historical stone structures, represents a very challenging matter due to several environmental and anthropogenic factors. Vicenza stone, a calcareous rock known for its historical significance and widespread use in architectural masterpieces, requires significant attention for conservation. In fact, as the demand for sustainable and effective preservation methods intensifies, the exploration of innovative consolidation strategies becomes essential. To this end, inorganic consolidants, based on alkaline silicate formulations and nano-silica, were explored for their promising performance in enhancing the surface properties and chemical stability of Vicenza stone. In particular, the durability of treated and untreated Vicenza stone samples was evaluated by means of accelerated weathering tests such as freeze–thaw cycles, salt crystallization and simulation of acid rain. The experimental results revealed that Vicenza stone is very resistant to the effects of freeze–thaw cycles and acid rain; both the accelerated weathering tests did not show significant differences between treated and untreated VS samples. A different behavior was detected for the test for resistance to salt crystallization, whose findings led us to deduce that, for this kind of degradation, it is possible to observe a more beneficial effect of the consolidation treatments on the stone durability.

## 1. Introduction

The conservation and preservation of natural stones is a critical area of study especially for historical and architectural heritage. One of the major challenges in modern conservation science is the design of suitable restoration projects combined with a focused selection of compatible materials [1,2,3]. In order to design the most effective and non-invasive conservation treatments, the durability of stones, which is greatly influenced by their intrinsic characteristics, such as texture, chemical and mineralogical composition, porosity degree and size, must be considered [4,5,6]. Also, several environmental factors, like air pollution, variations in temperature and humidity, water presence (liquid or vapor), and stone location and orientation, can strongly affect the resistance to decay and the degradation mechanisms of stones [4,7,8]. The most common deterioration mechanism of sedimentary stones in buildings involves the loss of intergranular cohesion due to the depletion of the natural interstitial binder [9], which is the final effect of a series of chemical–physical processes, leading to alterations in the chemical, physical and/or mechanical properties of the stone [9,10]. In this context, the use of consolidation treatments is essential both to re-establish cohesion within the damaged layer and to restore the original substrate physical–mechanical properties, but without altering the stone’s intrinsic features, such as water vapor permeability and aesthetic appearance [9,11,12]. The main sources of damage which can be addressed to prevent the degradation of natural porous stones in historic buildings are mostly related to the presence of water, and include freezing and thawing [13], salt crystallization [14,15], thermal and moisture expansion [16] and contact with water from rain or the ground [17]. These mechanisms often act in combination with each other and the final damage is a consequence of their complex interactions. In order to assess the durability of stone building materials, it is fundamental to perform weathering tests, which accelerate the natural decay process without altering its mechanism [14]. So, the evaluation of the level of damage to materials exposed to decay agents in laboratory conditions should allow us to estimate the degradation of the same material under real conditions [4,18,19].

This study deals with the durability of Vicenza stone (VS), a type of limestone that is especially renowned for its aesthetic appeal and workability, which has been extensively used in historical monuments and buildings in the Veneto region (Italy) [1,20]. As already discussed, VS is susceptible to weathering and degradation over time due to intrinsic and environmental factors, so the preservation of its structural integrity and aesthetic value requires the application of consolidation treatments. In recent decades, both organic and inorganic consolidating products were explored and, considering factors such as compatibility with the substrate, re-treatability, long-term efficacy and sustainability, the inorganic ones are the most widely used [9,21,22,23,24]. In particular, inorganic consolidants have been shown to be promising for their compatibility with calcareous stones and their ability to improve durability without significant changes to the appearance of the stones [21,23,25]. The performance of consolidation treatments with two alkaline silicates (lithium and sodium) and nano-silica on the main chemical–physical and morphological properties of VS have been preliminarily investigated [26]. On the basis of the positive results obtained, in the present investigation, the efficacy of these treatments on VS durability was assessed by means of accelerate weathering tests, consisting of freeze–thaw cycles, salt crystallization and simulation of acid rain.

## 2. Experimental

### 2.1. Materials

#### 2.1.1. Stone Material

**Figure 1:**
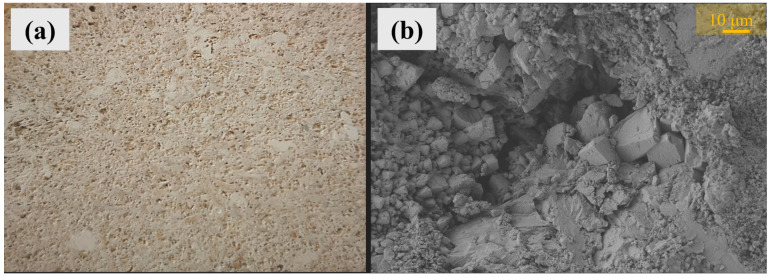
Macroscopic details (**a**) and SEM image (FESEM-EDS Merlin VP Carl Zeiss; magnification: 640×) (**b**) of Vicenza stone.

Vicenza stone (VS) was quarried from the Nanto quarry in the Berici Hills located near Vicenza, Italy. From the mineralogical point of view, it is a pale ivory limestone rock, primarily composed of calcite and dolomite with low silica and clay contents (CaCO_3_ > 90–95%) [1,27]. It originated from the Oligocene backreef zone biosparite formations and it is characterized by an abundance of bioclastic components, a distinctive feature of its texture [1,27]. The macroscopic and microscopic aspects of VS are reported in Figure 1. Moreover, considering that a very in-depth characterization of VS has already been reported elsewhere [26], a general overview of the main physical properties of VS is summarized in Table 1. All the VS samples tested in the present investigation were first rinsed with distilled water and then dried in an oven at 60 °C until a constant mass was achieved.

#### 2.1.2. Consolidant Products

Silica-based consolidants are probably the most commonly used commercial products for consolidation treatments on natural stones. In the present study, the impact of the consolidating treatments, using two alkaline silicates and a suspension of nano-silica, on Vicenza stone durability was thoroughly investigated. The alkaline silicates were a lithium silicate solution (LS) (Li_2_SiO_3_, SiO_2_: 20–25 wt%; Li_2_O: 2–3 wt%) and a sodium silicate solution (SS) (Na_2_SiO_3_, Na_2_O: 8.15 wt%, SiO_2_: 27.40 wt%), both supplied by Prochin Italia s.r.l. (Marcianise, Italy). The nano-silica-based suspension is a commercial product (Nano Estel, CTS s.r.l., Vicenza, Italy), which was provided by CTS s.r.l. (Altavilla Vicentina, Italy) and consists of an aqueous colloidal suspension (SiO_2_ content 30 wt%) with an average size of 10–20 nm, stabilized using sodium hydroxide (NaOH < 0.5%), with a resulting pH in the range of 9.8–10.4. Each consolidant was diluted with demineralized water (dilution of 1:1 by weight) prior to application to facilitate the penetration into the stone pores. The main chemical and physical properties of all consolidants and the procedure followed for the consolidation treatments are reported in detail elsewhere [26]. For all the consolidants, the treatments were carried out by immersion of the VS specimens at room temperature (20 ± 5 °C) in the diluted solutions for 60 min. After treatment, all specimens were stored in a climatic chamber (20 °C and 50% RH) for 10 days to ensure complete water evaporation and enhance the consolidating effect. The consolidated specimens were named throughout the text as “VS_NE”, “VS_LS” and “VS_SS” and were compared with untreated samples, labelled as “VS_REF”.

### 2.2. Durability Evaluation

Durability is one of the key aspects of the behavior of natural stones in the real environment. The durability of a rock can be defined as its resistance to alteration or decay when exposed to weathering agents, maintaining its main properties and aesthetic appearance over time and it represents an important parameter when assessing its quality as a building material [4,28]. Durability can be assessed using several approaches such as accelerated laboratory tests, complex environmental testing in test cabinets and exposure site testing [29]. In the present paper, the role of consolidation treatments on the durability of reference and treated VS samples was evaluated using accelerated weathering through freeze–thaw cycles, salt crystallization and simulation of acid rain (immersing samples in acidic solutions) [17,30].

#### 2.2.1. Freeze–Thaw Resistance

In order to determine the frost resistance of the treated and untreated specimens, freeze–thaw tests were performed according to UNI EN 12371 [31] on cubic specimens (5 × 5 × 5 cm^3^). The standard method was carried out by alternating cycles of freezing periods (6 h in air in a freezing tank) and thawing periods (6 h immersion in water). All the samples were subjected to 35 freeze–thaw cycles, starting from room temperature up to −12 °C. When the tests were completed, the deterioration of the samples was estimated by measuring their weight loss (Kern PBS620-3M, 0.01 g sensitivity, KERN & SOHN GmbH, Balingen, Germany) and P-wave velocity changes (Boviar DSP UTD 1004 Ultrasonic Device, 55 kHz transducers in direct arrangement, Boviar, Naples, Italy).

#### 2.2.2. Simulation of Acid Rain

**Figure 2:**
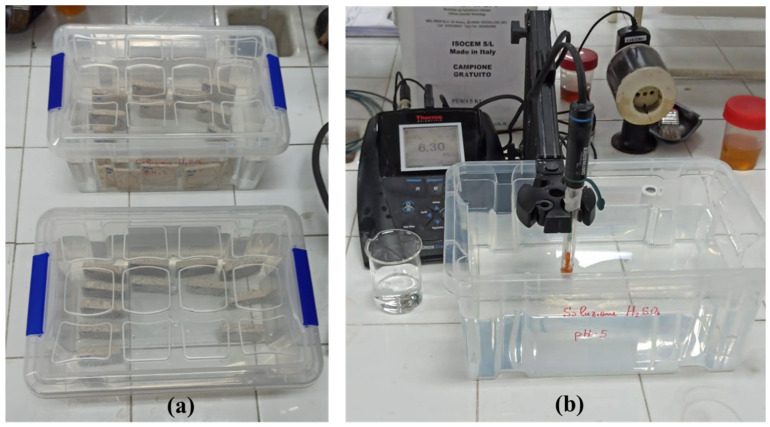
Simulation of acid rain test. (**a**) Containers with nitric and sulfuric acid solutions and (**b**) pH meter (Thermo Scientific, Monza, Italy, Orion Star A211) for pH adjustment during the test.

In order to assess the effect of acid rain, two acidic solutions (pH = 5) were prepared in distilled water: sulfuric acid (H_2_SO_4_, purchased from Carlo Erba, 96% analytical grade) and nitric acid (HNO_3_, supplied by J.T. Baker, 65% analytical grade). Sulfuric and nitric acids were selected considering that the acidity of real rain in recent years has been due to sulfuric acid (about 2/3) and nitric acid (1/3) [30,32]. This choice was also based on the continued expected relevance, in the future, of SO_x_- and NO_x_-induced rain acidification in many areas, particularly in developing countries, even though carbonic acid is expected to become more significant, especially in Europe, due to increasing CO_2_ emissions [30]. Additionally, most of the studies investigating the effects of simulated acid rain on carbonate stones used the above mentioned acids [17,30]. The pH of the simulated acid rain solutions was fixed at 5 because the pH value of real rain in European cities in recent years was estimated at around 4–5 [33]. Both treated and untreated samples were placed in a closed container with a volume of 5 L and immersed in the solutions (see Figure 2a). The tests were performed with five cycles of immersion, followed by drying phases according to methodology described by Eyssautier-Chuine et al. [17]. The first cycle was different from the others and consisted of 24 h of immersion before drying, while the following four involved 4 days of immersion before drying. The drying stage was the same for all the cycles and was performed by putting the samples at 45 °C for about 64 h. The pH of both solutions were adjusted twice a day (see Figure 2b) in order to maintain the initial pH of 5.0 because the reaction of carbonate stones in acidic environments tends to increase the pH.

The effects of the acidic weathering on the reference and treated VS samples were estimated at the end of every cycle by evaluation of visual alterations, weight loss and changes in surface color, which was evaluated using a CM-2500d Konica Minolta Spectrophotometer, Konica Minolta, Milan, Italy). Color measurements were carried out according to UNI EN 15886 [34] using the following experimental parameters: 8.0 mm-diameter viewing aperture, specular component included (SCI), illuminant D65 and 10° observer angle. The chromatic parameters were expressed according to the CIE (Commission Internationale d’Eclairage) L*a*b* space [35] and L*, a* and b* are, respectively, the lightness/darkness, the red/green and the yellow/blue coordinates. The color change (ΔE) was calculated according to the following equation:

ΔE = [(ΔL*)^2^ + (Δa*)^2^ + (Δb*)^2^]^1/2^

It is important to highlight that if ΔE < 3 [36], color variations are generally not perceived by human eye, while ΔE = 5 can be considered the maximum threshold value accepted as a chromatic alteration for treated stones [37].

Finally, after each cycle, 10 mL of both acid solutions was sampled, stored at 4 °C and analyzed by inductively coupled plasma atomic emission spectrometry (ICP-OES) (Optima 2100 DV ICP-OES, Perkin Elmer, Milan, Italy) in order to determine the variations in the concentrations of the major relevant elements (Ca, Si, Na and Li).

#### 2.2.3. Salt Crystallization Resistance

The assessment of the resistance of stones to salt crystallization was determined using the methodology outlined in the standard UNI EN 12370 [38], which was specifically designed for stones exhibiting a porosity exceeding 5%. These evaluations, often coupled by additional accelerated aging examinations (such as cyclic freezing and thawing), are commonly employed techniques for evaluating the extent of deterioration of stony materials when subjected to decay-inducing agents, in order to assess the overall durability of natural stones [14,39,40]. Notably, the formation of salts within porous structures is a significant factor in the decay of natural building stones, inducing extensive research in this area [21,41,42].

In this study, three cubic specimens (each with a side length of 5 cm) of Vicenza stone (both untreated and treated) were placed in a climatic chamber at a temperature of T = 20 °C and relative humidity Hr = 50% until a constant weight (*m*_0_) was achieved. Subsequently, the specimens were immersed in a sodium sulfate decahydrate solution (14% wt./wt.) for 2 h at T = 20 °C. Then, the specimens underwent gradual heating over 10 h to reach a temperature of 105 °C and they were kept at this temperature for 16 h. Finally, they were cooled to room temperature, stored for one day in the climatic chamber (20 °C, 50% Hr) and then weighed (*m_i_*). This cycle was repeated until either the specimens disintegrated or a maximum of 15 cycles was reached. After each cycle “i,” the change in mass (*m_v_*) was calculated by expressing the ratio of the weight after “i” cycles (*m_i_*) to the initial weight (*m*_0_).

$$m_{v}=\frac{m_{i}}{m_{0}}$$

The test outcomes were expressed through the aforementioned mass variation, the count of cycles required for specimen disaggregation and a photographic documentation were performed using a Dual LEICA 20 MP (Mono) f/1.6.

## 3. Results and Discussion

### 3.1. Freeze–Thaw Resistance

From visual inspection, the VS appeared resistant to freeze–thaw cycles; the examination of both the treated and reference specimens did not reveal any damage on the surface (cracks, rounding of corners, etc.). On the contrary, the evaluation of the weight losses after the freeze–thaw test revealed slight differences between the treated and untreated samples (see Table 2). In particular, all the consolidated samples exhibited similar weight losses, which were lower than that of the reference samples. This behavior could be related to the effect of the consolidation treatments, which led to a porosity reduction [26]. However, the evaluation of P-wave velocities before and after the freeze–thaw cycles (Table 1) further confirmed the trend showed by the weight losses. In fact, all the samples exhibited a slight reduction in P-wave velocities, an effect of the moderate deterioration induced by the weathering. As expected, this reduction was more evident for the untreated samples.

### 3.2. Simulation of the Effects of Acid Rain

**Figure 3:**
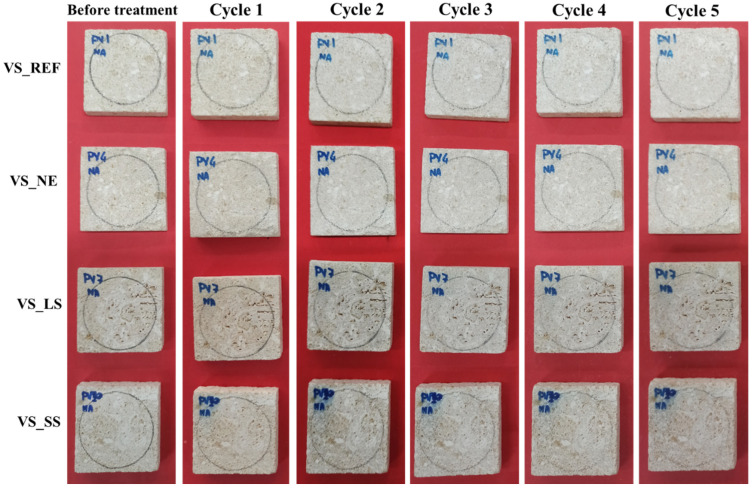
Photographic report of treated and untreated VS sample stones before and after the nitric acid test.

**Figure 4:**
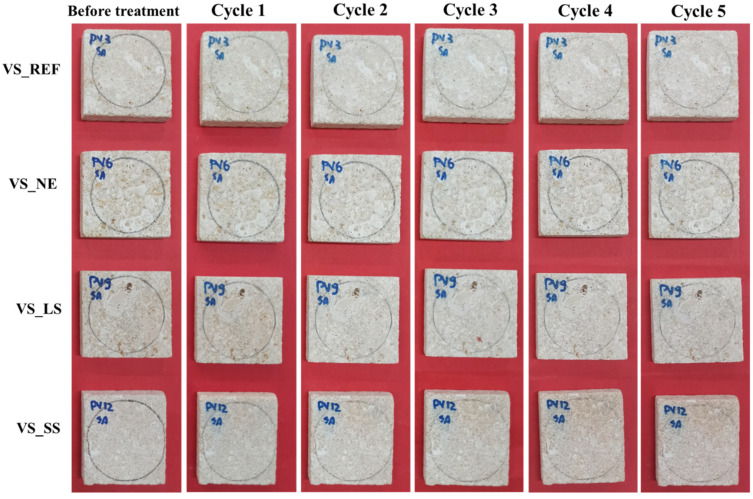
Photographic report of treated and untreated VS sample stones before and after the sulfuric acid test.

Photographic reports of the VS samples, before and after the acid tests, are shown in Figure 3 and Figure 4. The observations by the naked eye did not show clear changes in color or significant alterations in the stone surface for both acidic solutions and for all the VS samples that had undergone the weathering tests.

**Figure 5:**
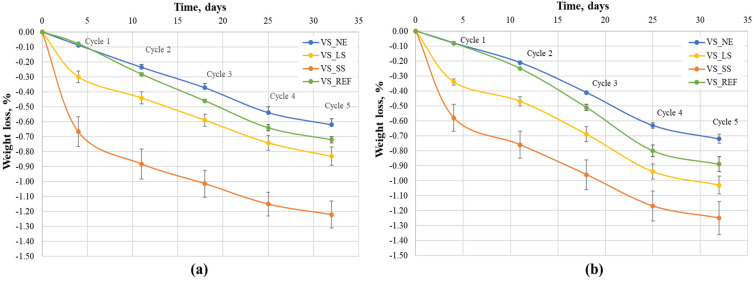
Weight loss versus time for all treated and untreated VS samples during the weathering tests in sulfuric acid (**a**) and nitric acid (**b**).

In order to evaluate the effects of the acidic weathering tests on all the samples, in Figure 5, their weight losses are reported and expressed as a percentage with respect to their initial weights. All the samples, both reference and treated, displayed a small decrease in weight over time and followed a similar trend for both acidic treatments. To better understand the trends of the above-mentioned curves, it is important to highlight some differences between the consolidation mechanisms in the samples. For all the treatments, the consolidation effect was due to a water-solvent evaporation mechanism which led to the bonding of silica particles, creating a silica gel within the stone pores [43]. Moreover, the use of alkaline silicates caused the formation of additional alkaline-based compounds, mainly carbonate species [26].

It is worth noting that the samples treated with alkaline silicates showed greater weight losses compared to both the VS_REF and VS_NE samples. This unexpected behavior could be related to the different solubilities of the above-mentioned carbonate species and silica gel. In particular, sodium carbonate exhibits a solubility (220 g/L, 25 °C) that is significantly higher than that of lithium carbonate (13.3 g/L, 20 °C), while silica gel is characterized by a very low solubility. Therefore, the evident weight losses after the first cycle (see Figure 5) for both VS_LS and VS_SS are related to the dissolution of lithium and sodium carbonates, respectively. Moreover, by inspecting the weight losses of all the samples after the first cycle, all the curves exhibited a similar trend due to the decay of calcareous stone when in contact with acidic solutions, which occurred in all of the samples.

**Figure 6:**
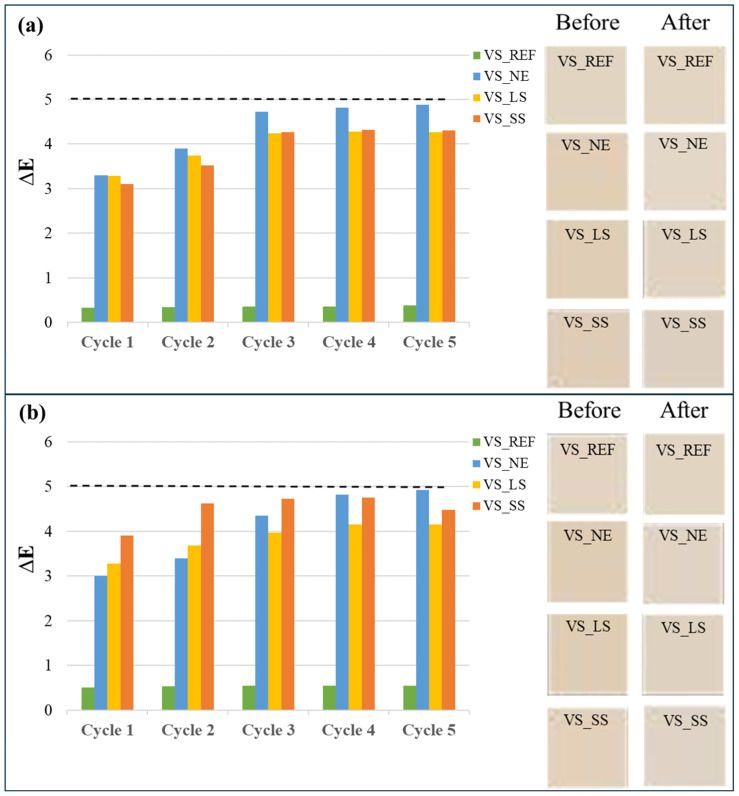
Changes in total color differences (ΔE) with relative chromatic variations for all VS samples after the weathering tests in sulfuric (**a**) and nitric (**b**) acids.

Changes in surface color after the weathering tests are reported in Figure 6 and Table 3. The results showed that the VS_REF samples did not exhibit significant color variations after being in contact with both acidic solutions. On the other hand, all the consolidated samples presented surface color changes with ΔE values ranging between 4 and 5 at the end of the tests. In particular, the acidic weathering in both acidic solutions led to a moderate lightening of all the samples (see Figure 6), which was reflected by the ΔL* positive values (Table 3). This result could be related to the effects of weathering that remove the chromatic effects of the previous consolidating treatments, which led to a darkening of the surface [26]. Finally, it is worth noting that most of the chromatic variations occurred during the first cycle of the tests, confirming the trend exhibited by the weight losses.

**Figure 7:**
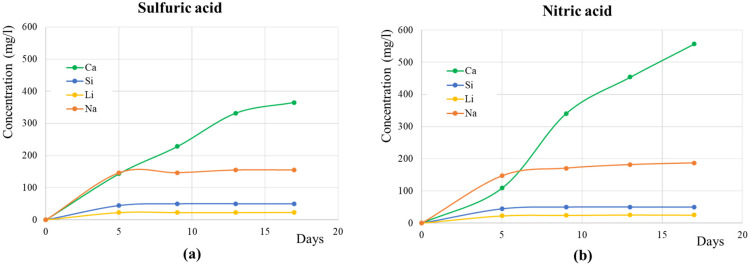
Variation in the main cation concentrations (cumulative) during the weathering tests in sulfuric (**a**) and nitric (**b**) acids.

In order to monitor the effects of the acidic solutions on the decay of the VS samples, the concentrations of Ca, Si, Na and Li in the immersion solutions as a function of time are reported in Figure 7. For each cycle, the concentration values reported are the cumulative values since the start of the experiment.

The concentration of Ca^2+^ increased with time for both weathering tests. In particular, it reached about 350 and 550 mg L^−1^ at the end of the test immersion in sulfuric and nitric acids, respectively, revealing that the test with HNO_3_ seemed to be a little more aggressive (higher Ca^2+^ concentrations) than the H_2_SO_4_ one. Regarding the other cations, which can be considered to be more related to the presence of the consolidants, their concentrations were basically the same in both acidic solutions. It is worth noting that Na^+^ exhibited higher concentrations, as a consequence of the more significant dissolution of sodium carbonates (see Figure 5). Moreover, the most significant dissolution happened during the first cycle (after 5 days), confirming the trend shown by the other parameters (weight loss and color changes). In fact, except for Ca^2+^, after 5 days, all the concentration values did not change significantly, almost reaching the maximum value.

### 3.3. Resistance to Salt Crystallization

**Figure 8:**
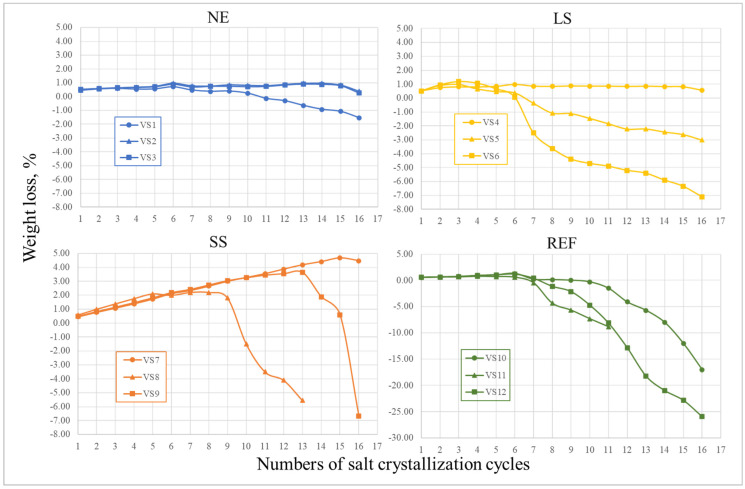
Salt crystallization resistance results for VS_NE, VS_LS, VS_SS and VS_REF samples.

**Figure 9:**
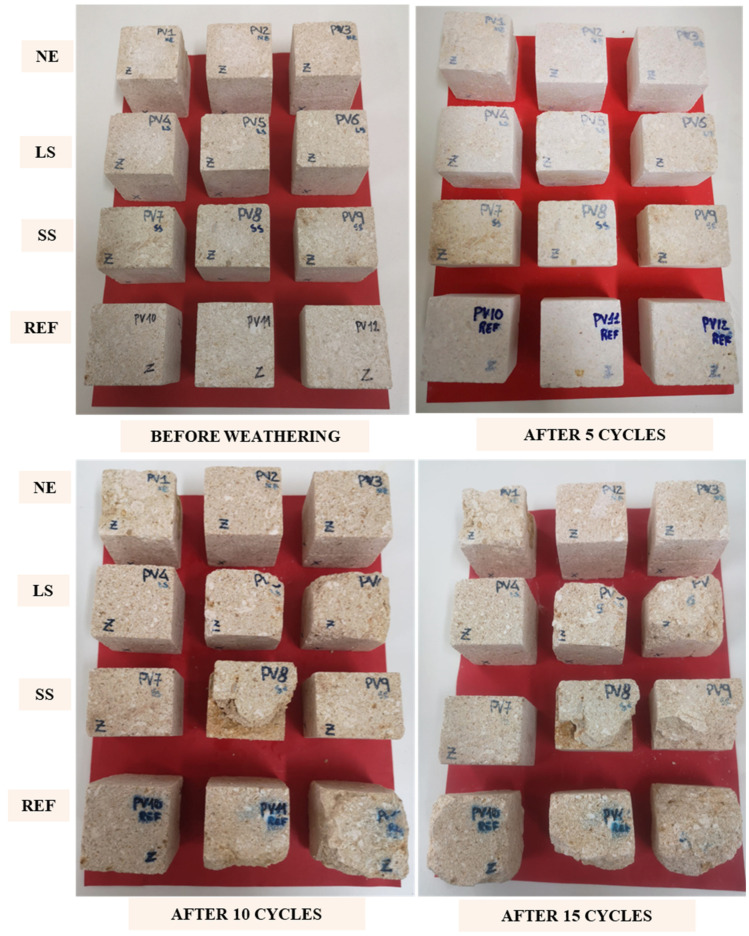
Photographic record of VS samples after 5, 10 and 15 cycles of salt crystallization.

The salt crystallization test was performed on the reference and treated samples, and both the weight loss and a photographic record after 5, 10 and 15 cycles are reported, respectively, in Figure 8 and Figure 9. The results showed that none of the samples exhibited evident decay or weight loss until the fifth cycle; on the contrary, a slight weight increase was detected due to the salt crystallization. With further weathering, a different trend was noticed. In particular, all the VS_REF samples were strongly affected by salt crystallization, as they underwent significant weight losses and shape alterations (see Figure 9). In particular, the VS_REF11 sample broke after 9 cycles and VS_REF12 presented, after 15 cycles, the highest weight loss (≈−25%, see Figure 8). Regarding the treated samples, the results were more heterogeneous: all the weight losses were lower than the reference ones, but some samples were damaged despite the consolidating treatment.

As already observed after the weathering test in acidic solutions, the more interesting results were obtained with the Nano Estel treatment (VS_NE samples). In fact, two of the three tested samples (VS_NE2 and VS_NE3) did not show any significant weight loss or evident shape alteration (see Figure 9), while the third sample (VS_NE1) only exhibited a slight weight loss (≈−1.5%).

On the contrary, the treatment with alkaline silicates gave rise to more discordant results. In fact, some of the treated samples were strongly affected by salt crystallization, with evident decay, while others underwent the weathering treatment without any evident damage (VS_SS7 and VS_LS4). It is worth noting that the heterogeneity of the obtained results could be related to the heterogeneity of the Vicenza stone. More specifically, the salt crystallization inside the stone pores led to a volume increase, which caused internal stresses and the possible formation of microcracks. Such defects, under unpredictable conditions, can propagate, causing the brittle fracture of the samples.

## 4. Conclusions

Natural stones have always played a significant role in the construction of historical buildings and monuments. For this reason, their weathering represents a serious concern because it may cause a rapid change in the initial petrophysical and chemical properties of the rocks and thus limit their durability. In particular, the preservation of calcareous stones is a significant issue because of the widespread utilization of this type of stone in cultural heritage sites. In fact, stones like marble, limestone and calcareous sandstone, which consist mostly of carbonate minerals, can be highly susceptible to various forms of deterioration, especially because carbonates species are often the most soluble components in building materials. Thus, the durability of Vicenza stone, treated with inorganic silica-based consolidants (two alkaline silicates and an aqueous suspension of nanometric silica), was explored by means of accelerated weathering tests. The moderate deterioration induced by freeze–thaw cycles and by simulation of the effects of acid rain led to the conclusion that Vicenza stone is resistant to both of these degradation mechanisms, showing only slight differences in the behaviors of the reference and consolidated samples. On the contrary, the beneficial effects of the consolidation treatments were much more evident in the resistance to salt crystallization. In fact, while all the untreated Vicenza stone samples exhibited very significant weight losses and shape alterations, the treated samples showed better behaviors, even if the results were much more heterogeneous. In particular, the consolidation treatment performed by means of nano-sized silica particles was the most effective, probably due to the different porosimetric structure created, characterized by a greater pore size distribution and pore volume (>10 μm) (see [26]), which should lead to greater durability on the basis of pore structure estimators for stone durability [41].

So, the above-discussed findings indicate that the use of silica-based consolidants (particularly nano-silica) on calcareous stones can be proposed as a valid alternative to those based on alkaline hydroxides.

Finally, it is important to underline that these kind of studies focused on the degradation of natural geological materials are crucial to provide insights into the physical–chemical processes which cause the deterioration of heritage sites and historical monuments over time and, most of all, are essential for developing effective conservation strategies. So, the dissemination of this knowledge both to the experts in the field and to a wider public are vital to ensuring the long-term conservation and restoration of historical and cultural assets, preserving them for future generations.

## Figures and Tables

**Table 1:** Summary of the main physical properties of Vicenza stone [26].

Property	
Apparent density (g/cm^3^)	1.99 ± 0.02
Open porosity (%)	26.8 ± 0.9
Water absorption (%)	13.5 ± 0.6
Capillary absorption coefficient (mg/cm^2^ s^−1/2^)	11.8 ± 0.83
MIP porosity (m^2^/g)	1.16 ± 0.09

**Table 2:** Weight losses and P-wave velocities of VS samples after freeze–thaw cycles.

Sample	Weight Loss (%)	UPV (m/s) Before Weathering	UPV (m/s) After Weathering
VS_REF	4.4 ± 1.0	3348 ± 31	3189 ± 39
VS_NE	0.9 ± 0.5	3438 ± 23	3390 ± 27
VS_LS	1.1 ± 0.4	3483 ± 35	3410 ± 31
VS_SS	1.2 ± 0.4	3581 ± 28	3525 ± 33

**Table 3:** Variations in the chromatic parameters after the weathering tests in sulfuric (SA) and nitric (NA) acidic solutions.

SA	Before Weathering			After 5 Cycles			
Sample	L*	a*	b*	L*	a*	b*	∆E
VS_REF	85.89	1.72	11.24	86.26	1.67	11.17	0.38
VS_NE	83.73	2.64	13.61	86.41	1.65	9.44	4.89
VS_LS	83.31	2.93	13.82	84.69	1.95	10.10	4.27
VS_SS	83.20	3.26	13.13	83.82	2.08	8.99	4.30
**NA**	**Before Weathering**			**After 5 Cycles**			
**Sample**	L*	a*	b*	L*	a*	b*	**∆E**
VS_REF	85.65	2.19	11.17	85.89	1.72	11.01	0.55
VS_NE	82.96	3.09	13.73	85.29	1.84	9.39	4.93
VS_LS	83.08	2.87	14.20	84.62	1.81	10.50	4.15
VS_SS	84.31	2.89	13.16	85.19	1.60	8.36	4.48

## Data Availability

Dataset available on request from the authors.
